# Real-World-Time Simulation of Memory Consolidation in a Large-Scale Cerebellar Model

**DOI:** 10.3389/fnana.2016.00021

**Published:** 2016-03-03

**Authors:** Masato Gosui, Tadashi Yamazaki

**Affiliations:** ^1^Department of Communication Engineering and Informatics, Graduate School of Informatics and Engineering, The University of Electro-CommunicationsTokyo, Japan; ^2^Neuroinformatics Japan Center, RIKEN Brain Science InstituteSaitama, Japan; ^3^Artificial Intelligence Research Center, National Institute of Advanced Industrial Science and TechnologyIbaraki, Japan

**Keywords:** cerebellum, model, memory consolidation, optokinetic response, realtime simulation, graphics processing unit

## Abstract

We report development of a large-scale spiking network model of the cerebellum composed of more than 1 million neurons. The model is implemented on graphics processing units (GPUs), which are dedicated hardware for parallel computing. Using 4 GPUs simultaneously, we achieve realtime simulation, in which computer simulation of cerebellar activity for 1 s completes within 1 s in the real-world time, with temporal resolution of 1 ms. This allows us to carry out a very long-term computer simulation of cerebellar activity in a practical time with millisecond temporal resolution. Using the model, we carry out computer simulation of long-term gain adaptation of optokinetic response (OKR) eye movements for 5 days aimed to study the neural mechanisms of posttraining memory consolidation. The simulation results are consistent with animal experiments and our theory of posttraining memory consolidation. These results suggest that realtime computing provides a useful means to study a very slow neural process such as memory consolidation in the brain.

## 1. Introduction

Memory formation has two stages: memory acquisition and memory consolidation (Dudai, 2004). A single session of training forms a type of memory which is fragile and persists only a short period up to minutes to hours. This phase is called memory acquisition. After the training, the learned memory, a short-term memory, decays spontaneously and quickly within a day. Meanwhile, repeated training with a sufficient rest between training sessions gradually form another type of memory, a long-term memory, which is robust and persists for days and weeks. This phase is called memory consolidation. Memory consolidation occurs after training but not during training. That is, when we take a rest after training, the brain still continues working to consolidate the learned memory. This posttraining memory consolidation is thought to be the basis of spacing effect (Ebbinghaus, 1885), in which a massed training is inferior to repeated training to form a robust long-term memory, even if the total training time is equal. Therefore, it is important to study how the brain works after training as well as during training to elucidate the memory mechanisms and our behaviors.

In cerebellar motor learning, both memory formation and consolidation occur within the cerebellum. In gain adaptation of vestibulo-ocular reflex (VOR) and optokinetic response (OKR), parallel fiber-Purkinje cell (PF-PC) synapses in the cerebellar cortex store short-term memory, whereas mossy fiber-vestibular nuclear cell (MF-VN) synapses in the brain stem store long-term memory (Kassardjian et al., 2005; Shutoh et al., 2006). OKR is an oculomotor reflex in which the eye moves to the same direction of the visual world's movement to reduce the slip of the retinal image. In OKR adaptation, the amplitude of eye movement, called gain, changes by training. By a single 1-h training, the gain increases quickly, which corresponds to memory acquisition. After the training, the gain decreases naturally to the original level within a day. By repeating the 1-h training everyday, the gain increases gradually throughout 1 week (Shutoh et al., 2006), which represents memory consolidation. Moreover, injection of muscimol, a γ-Aminobutyric acid (GABA) receptor agonist, to the cerebellar cortex immediately after the training disrupts memory consolidation (Okamoto et al., 2011), indicating that training alone is not sufficient for memory consolidation. Accumulating evidence suggests that posttraining memory consolidation of OKR gain takes the following steps (Shutoh et al., 2006). By a single 1-h training, PF-PC synapses undergo LTD induced by conjunctive activation of PFs and the CF innervated to the same PCs (Ito, 1989), and thereby the OKR gain increases. After the training, PFs gradually recover from the LTD, which erase the memory of learned OKR gain in the cortex. On the other hand, because inhibition exerted by PCs to VN is weakened due to the LTD, the VN is deporalized tonically. This deporalization, combined with presynaptic MF activation, induces LTP at MF-VN synapses (McElvain et al., 2010; Person and Raman, 2010), and thereby forming the memory of OKR gain in the nucleus. In this way, while the cortical memory is erased gradually after the training, the nuclear memory forms simultaneously as a long-term memory, as if the learned cortical memory is transferred to the nucleus and consolidated there.

We have proposed a theory of the cerebellar posttraining memory consolidation in OKR adaptation (Yamazaki et al., 2015). The theory captures an essence of the macroscopic dynamics of synaptic mechanisms underlying the posttraining memory consolidation. On the other hand, the theory does not provide insights on mesoscopic cellular/synaptic dynamics on the posttraining memory consolidation. For example, the theory does not tell us about spatiotemporal spike patterns of individual neurons. To study the detailed cellular/synaptic dynamics, an elaborated, realistic cerebellar model is necessary. A problem of such elaborated models, however, is that they would spend too much computational time. Typically, computer simulation of large-scale spiking network models is 10–100 times slower than the real-world time (Nageswaran et al., 2009). This means, if we wanted to carry out a computer simulation of memory consolidation for 1 week, and the computer simulation was 100 times slower than real time, the simulation would spend about 2 years in total to complete. This is practically impossible.

In this study, we adopted high-performance computing (HPC) technology to solve these problems. We used graphics processing units (GPUs) to calculate equations of neurons in parallel, which could speed up the numerical simulation drastically. Specifically, we built a very large-scale spiking network model of the cerebellum composed of 1 million neurons, which is a model of 1 mm^3^ of cats' cerebellum. Moreover, owing to the parallel computing on GPUs, we were able to conduct the computer simulation fast enough to complete a very long computer simulation in a practical time, Eventually, we achieved realtime simulation, which means that computer simulation of the cerebellar activity for 1 s completes within 1 s in the real-world time (Igarashi et al., 2011; Yamazaki and Igarashi, 2013). This is essential for computer simulation of the cerebellar posttraining memory consolidation, because the memory consolidation takes days or even weeks. Using the present cerebellar model, we performed computer simulation of long-term OKR adaptation of training for 5 days, and obtained qualitatively the same results with experiments (Shutoh et al., 2006) and our previous theoretical model (Yamazaki et al., 2015). We also examined the detailed spike patterns of neurons, which was abstracted and therefore ignored in our theory.

## 2. Materials and methods

### 2.1. Model

**Figure 1:**
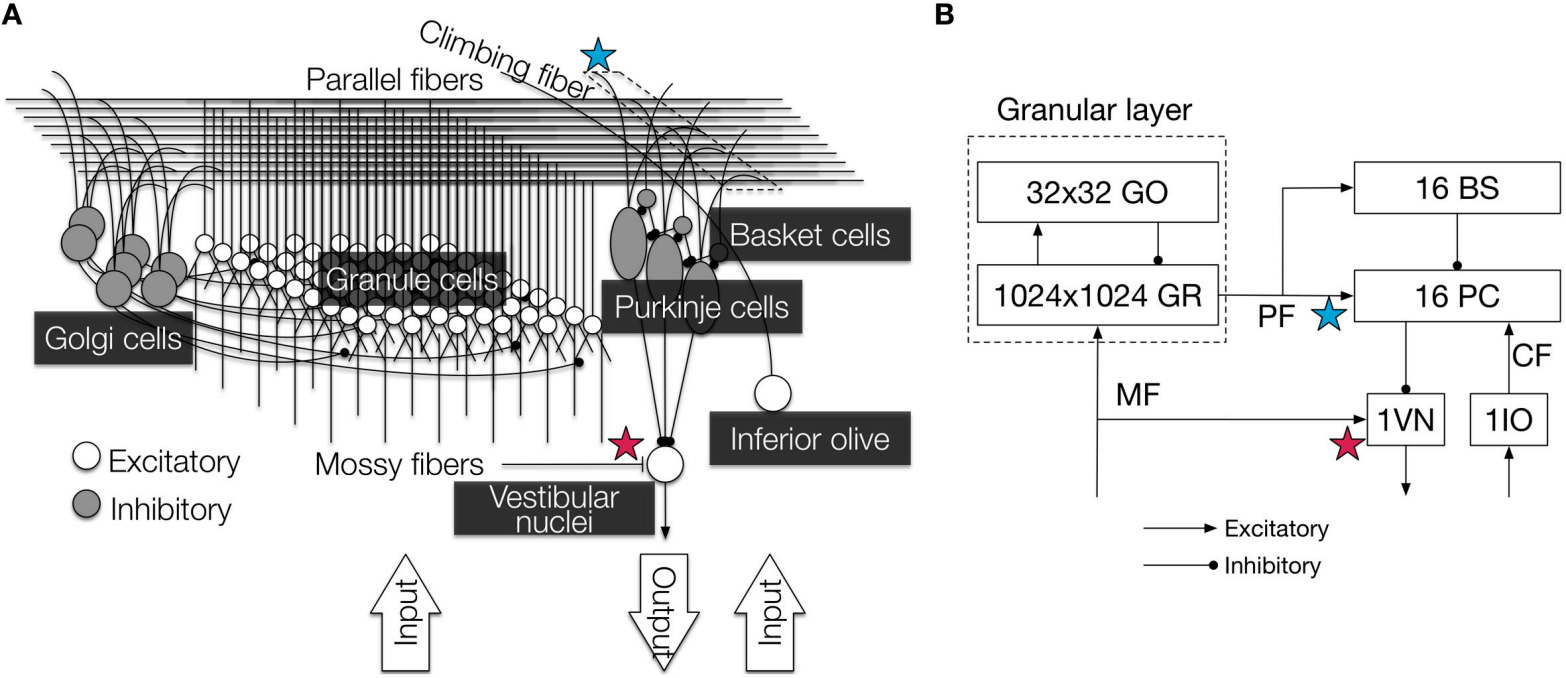
**Illustration of the cerebellar circuit implemented in this study**. **(A)** Detailed 3D diagram with locations of synaptic plasticity. We implemented six major types of neurons: granule cells, Golgi cells, PCs, basket cells, inferior olivary cell, and VN. They were connected according to anatomical data, and cell parameters were taken from electrophysiological data. Contextual information and error information were conveyed by MFs and a CF, respectively, and the VN provided the final output of the circuit. PF-PC synapses (blue star) and MF-VN synapses (red star) underwent plastic change. The figure was reproduced from Yamazaki and Igarashi (2013). **(B)** 2D diagram of the connectivity and the number of neurons. PF-PC synapses (blue star) and MF-VN synapses (red star) underwent plastic change as in **(A)**. Arroheads represent types of synaptic connections (triangle, excitatory; circle, inhibitory). GR, granule cell; GO, Golgi cell; PC, Purkinje cell; BS, basket cell; VN, vestibular nucleus; IO, inferior olive; MF, mossy fiber; CF, climbing fiber; PF, parallel fiber.

Our cerebellar model is built based on a 1 mm^3^ of the cerebellar corticonuclear microcomplex (Figure 1) of cats, which is thought to be a functional module of the cerebellum (Ito, 1984, 2012). The original model had 100,000 granule cells, which is 10 times smaller than cats' cerebellum (Ito, 1984), and was already reported elsewhere (Yamazaki and Tanaka, 2007; Yamazaki and Nagao, 2012; Yamazaki and Igarashi, 2013). In this study, we extended the previous model as follows. First, the present model includes 1 million granule cells, thereby the model includes the same number of neurons with 1mm^3^ of the cats' cerebellum. Second, the present model has synaptic plasticity at mossy fiber-vestibular nuclear cell (MF-VN) synapses, as well as parallel fiber-Purkinje cell (PF-PC) synapses. Except the number of granule cells and MF-VN synaptic plasticity, the previous and present models are the same. Therefore, we summarize the model specification only briefly below. The details are found in our previous papers (Yamazaki and Tanaka, 2007; Yamazaki and Nagao, 2012; Yamazaki and Igarashi, 2013).

The present model is composed of 1,048,576 (=1024 × 1024) granule cells, 1024 Golgi cells, 16 PCs, 16 basket cells, 1 inferior olivary cell, and 1 VN, connected according to cats anatomical data (Yamazaki and Tanaka, 2007). Neurons are modeled as conductance-based integrate-and-fire units (Gerstner et al., 2014):

(1) $$\begin{array}{l} C\frac{du}{dt}=-g_{\text{leak}}\left(u\left(t\right)-E_{\text{leak}}\right)-g_{\text{ex:AMPA}}\left(t\right)\left(u\left(t\right)-E_{\text{ex}}\right)\\ -\;g_{\text{ex:NMDA}}\left(t\right)\left(u\left(t\right)-E_{\text{ex}}\right)-g_{\text{inh:GABA}}\left(t\right)\left(u\left(t\right)-E_{\text{inh}}\right)\\ -\;g_{\text{ahp}}\left(t\right)\left(u\left(t\right)-E_{\text{ahp}}\right)+I_{\text{ext}}\left(t\right), \end{array}$$

where *u*(*t*) is the membrane potential at time *t*, *C* is the capacitance, *g*_leak_ and *E*_leak_ are the conductance and reversal potential of the leak current, respectively, *g*_ex:AMPA_(*t*), *g*_ex:NMDA_(*t*), *g*_inh:GABA_(*t*) are synaptic conductances of excitatory α-amino-3-hydroxy-5-methyl-4-isoxazolepropionic acid (AMPA) and *N*-methyl-*D*-aspartic acid (NMDA), and inhibitory GABA synapses, *E*_ex_ and *E*_inh_ are reversal potentials, *g*_ahp_(*t*) and *E*_ahp_ are the conductance and reversal potential of after-hyper polarization, respectively, and *I*_ext_(*t*) is an external current. When *u*(*t*) exceeds a threshold θ_spike_ at time *t*, the neuron elicits a spike at time *t*. Cell parameters are taken from turtles and rodents electrophysiological data (Yamazaki and Tanaka, 2007). The values used in this study are summarized in Table 1. Synaptic conductance *g*_*x*_(*t*) for type *x* is calculated as a convolution of presynaptic spike events with an exponential kernel as

(2) $$\begin{array}{l} g_{x}\left(t\right)=\underset{j}{\sum }w_{j}\cdot {ḡ}_{x}\underset{f\in S_{j}}{\sum }\exp_{x}\left(-\left(t-t^{\left(f\right)}\right)\right)\Theta \left(t-t^{\left(f\right)}\right), \end{array}$$

where ḡ_*x*_ is the peak conductance, *w*_*j*_ is the synaptic weight which is constant, *S*_*j*_ is the set of spikes elicited by presynaptic cell *j*, *t*^(*f*)^ is the spike time for the *f*th spike, exp_*x*_(*t*) is the exponential kernel, and Θ(*t*) is the Heaviside step function. The exponential kernels used in the present study are summarized in Table 2, whereas the synaptic weights are shown in Table 3.

**Table 1:** **Summary of cell parameters**.

**Parameter**	**Neuron type**					
	**GR**	**GO**	**PC**	**BS**	**VN**	**IO**
θ_spike_ (mV)	−35.0	−52.0	−55.0	−55.0	−38.8	−50.0
*C* (pF)	3.1	28.0	107.0	107.0	122.3	10.0
*g*_leak_ (nS)	0.43	2.3	2.32	2.32	1.63	0.67
*E*_leak_ (mV)	−58.0	−55.0	−68.0	−68.0	−56.0	−60.0
ḡ_ex:AMPA_ (nS)	0.18	45.5	0.7	0.7	50.0	1.0
ḡ_ex:NMDA_ (nS)	0.025	30.0	-	-	25.8	-
*E*_ex_ (mV)	0	0	0	0	0	0
ḡ_inh_ (nS)	0.028	-	1.0	-	30.0	0.18
*E*_inh_ (mV)	−82.0	-	−75.0	-	−88.0	−75.0
ḡ_ahp_ (nS)	1.0	20.0	0.1	0.1	50.0	1.0
*E*_ahp_ (mV)	−82.0	−72.7	−70.0	−70.0	−70.0	−75.0
τ_ahp_ (ms)	5.0	5.0	5.0	2.5	2.5	10.0
*I*_ext_ (nA)	-	-	0.25	0.1	0.8	−

*GR, granule cell; GO, Golgi cell; PC, Purkinje cell; BS, basket cell; VN, vestibular nuclear neuron; IO, inferior olivary neuron; -, nonexistent*.

**Table 2:** **Summary of synaptic functions**.

**Neuron type**	**Equation**
GR	exp_ex:AMPA_(*t*) = *e*^−*t*∕1.2^
	exp_ex:NMDA_(*t*) = *e*^−*t*∕52.0^
	exp_inh_(*t*) = 0.43 × *e*^−*t*∕7.0^+0.57 × *e*^−*t*∕59.0^
GO	exp_ex:AMPA_(*t*) = *e*^−*t*∕1.5^
	exp_ex:NMDA_(*t*) = 0.33 × *e*^−*t*∕31.0^+0.67 × *e*^−*t*∕170.0^
PC	exp_ex:AMPA_(*t*) = *e*^−*t*∕8.3^
	exp_inh_(*t*) = *e*^−*t*∕10.0^
BS	exp_ex:AMPA_(*t*) = *e*^−*t*∕8.3^
VN	exp_ex:AMPA_(*t*) = *e*^−*t*∕9.9^
	exp_ex:NMDA_(*t*) = *e*^−*t*∕30.6^
	exp_inh_(*t*) = *e*^−*t*∕42.3^
IO	exp_ex:AMPA_(*t*) = *e*^−*t*∕10.0^

*Abbreviations as in Table 1*.

**Table 3:** **Summary of synaptic weights**.

**Presynaptic neuron**	**Postsynaptic neuron**					
	**MF**	**GR**	**GO**	**PC**	**BS**	**VN**
MF	-	4.0	-	-	-	0.2
GR	-	-	0.00004	0.00075	0.00015	-
GO	-	10.0	-	-	-	-
PC	-	-	-	-	-	0.05
BS	-	-	-	5.3	-	-
IO	-	-	-	1.0	-	-

*Abbreviations as in Table 1*.

The model has two distinct synaptic plasticity sites. One is PF-PC synapses, which undergo long-term depression (LTD) by conjunctive activation of granule-cell axons called parallel fibers (PFs) and a climbing fiber (CF) innervating to the same PC (Ito, 1989), and long-term potentiation (LTP) as well by sole activation of PFs (Lev-Ram et al., 2003). We modeled these bidirectional plasticity as follows:

(3) $$\begin{array}{l} \tau_{w}\frac{dw_{ij}}{dt}=-w_{ij}\left(t\right)+x_{ij}\left(t\right)\\ \tau_{x}\frac{dx_{ij}}{dt}=-x_{ij}\left(t\right)-c_{\text{LTD}}\overset{50\text{ms}}{\underset{s=0}{\sum }}{\text{PF}}_{ij}\left(t-s\right)\text{CF}\left(t\right)+c_{\text{LTP}}{\text{PF}}_{ij}\left(t\right), \end{array}$$

where *w*_*ij*_(*t*) is the synaptic weight between PC *i* and PF *j*, τ_*w*_ and τ_*x*_ are time constants where τ_*w*_ ≪ τ_*x*_, *x*_*ij*_(*t*) is an internal variable, *c*_LTD_ and *c*_LTP_ are constants, PF_*ij*_(*t*) is 1 if PF *j* on PC *i* elicits a spike at time *t* and 0 otherwise, and CF(*t*) is 1 if the climbing fiber elicits a spike at time *t* and 0 otherwise. The term $\overset{50\text{ms}}{\underset{s=0}{\sum }}{\text{PF}}_{ij}\left(t-s\right)\text{CF}\left(t\right)$ means that PFs that elicit spikes 0–50 ms earlier than the time when the climbing fiber elicits a spike undergo LTD. If *n* spikes travel along a PF during 50 ms, the weight change becomes *n* times *c*_LTD_. Transmission delay of PF spikes might be essential for plasticity (Knoblauch et al., 2014). The conduction velocity of PFs has been experimentally estimated as 0.24 m/s (Vranesic et al., 1994). This results in the transmission delay of 1 mm PF is maximally about 4.2 ms, which could be negligible as long as we assume 50 ms time window for LTD. Therefore, we did not model transmission delays of PF spikes. On the other hand, we do not exactly describe the biological counterpart of *x*_*ij*_. A potential interpretation of *x*_*ij*_ would be intracellular concentration of some kinases involving PKC-MAPK positive feedback loop, which plays an essential role in maintenance of induced LTD (Kuroda et al., 2001). The initial values of *w* and *x* were set at 1.0 and 0.0, respectively.

The other plasticity is MF-VN synapses, which undergo bidirectional plasticity by a modified Hebbian mechanism. The original equation was proposed by our previous theoretical model (Yamazaki et al., 2015) based on Zhang and Linden (2006); Person and Raman (2010); McElvain et al. (2010) as follows:

(4) $$\begin{array}{l} \tau_{v}\frac{dv}{dt}=-v\left(t\right)\langle \text{MF}\left(t\right)\rangle +\langle \text{MF}\left(t\right)\left(\text{VN}\left(t\right)-\theta \left(t\right)\right)\rangle , \end{array}$$

where τ_*v*_ is time constant, *v*(*t*) is the synaptic weight at MF-VN synapses at time *t*, MF(*t*) is the activity of MFs, VN(*t*) is the activity of VN, 〈·〉 is the temporal average over a certain time window (we assumed 6 s), and θ(*t*) is a running average of VN(*t*), namely θ(*t*) = 〈VN(*t*)〉. The left-hand side represents the temporal increment of *v*(*t*). The 1st term in the right-hand side represents LTD by sole activation of MFs, and 2nd term represents the Hebbian mechanism, where the weight change correlates with the correlated activity of pre- and postsynaptic neurons. Here, the term θ(*t*) acts as a threshold; only when the postsynaptic neuron is activated strongly to exceed θ(*t*), the synapses undergo LTP, otherwise LTD or no change. In this way, θ(*t*) determines the direction of synaptic change. Moreover, the value of θ(*t*) itself changes temporally depending on the temporal history of VN(*t*). Higher θ(*t*) value makes the synapse harder to undergo LTP. The initial value of *v* was set at 1.0. The parameters for *w* and *v* are summarized in Table 4.

**Table 4:** **Summary of learning parameters**.

**Parameter**	**Value**
τ_*w*_ (min)	20.0
τ_*x*_ (min)	240.0
*c*_LTD_	0.005
*c*_LTP_	0.1

As far as we have tested, the general network dynamics does not change so largely over a wide range of parameter settings. We have found three points that are necessary to achieve robust learning. First, granule-Golgi cell recurrent network should be tuned so as to generate the population code of granule cells robustly. Second, basket cell → PC synaptic connections should not be so strong; otherwise, PCs would be silent completely. Third, PC → VN synaptic connections should not be so strong; otherwise, VN would be silent completely. If we satisfy these three points, the network, as far as we have tested, works robustly.

### 2.2. Simulation paradigm

We conducted computer simulation of long-term OKR gain adaptation as in Shutoh et al. (2006). Specifically, we repeated a 1-h simulated OKR training followed by 23-h rest 5 times corresponding to 5-days training. In each OKR training, simulated optokinetic stimulus is fed to MFs, and retinal slip is fed to a CF. Both optokinetic stimulus and retinal slip are modeled as Poisson spikes with the following firing rate:

(5) $$\begin{array}{l} f_{{\text{MF}}_{\text{train}}}\left(t\right)=\bar{{\text{MF}}_{\text{train}}}\left(1+\sin\frac{2\pi t}{T}\right)\;\left(\text{for MFs}\right)\\ f_{{\text{CF}}_{\text{train}}}\left(t\right)=\bar{{\text{CF}}_{\text{train}}}\left(1+\sin\frac{2\pi t}{T}\right)\;\left(\text{for a CF}\right), \end{array}$$

where *f*_MF_train__(*t*) and *f*_CF_train__(*t*) are the firing rate of MFs and a CF, respectively, $\bar{{\text{MF}}_{\text{train}}}$ and $\bar{{\text{CF}}_{\text{train}}}$ are the mean activity of MFs and a CF, which are set at 15 spikes/s and 1.5 spikes/s, respectively. *T* is a period of a cycle of optokinetic stimulus, which is assumed to be rotated sinusoidally in front of animal subjects. We set *T* = 6 s consistently with the experiments (Shutoh et al., 2006). Because one cycle is 6 s, daily 1-h training consists of 600 cycles of simulated optokinetic stimulus. On the other hand, after training, we assumed that both MFs and a CF elicited spikes spontaneously with the following firing rate:

(6) $$\begin{array}{l} f_{{\text{MF}}_{\text{rest}}}\left(t\right)=\bar{{\text{MF}}_{\text{rest}}}\;\left(\text{for MFs}\right)\\ f_{{\text{CF}}_{\text{rest}}}\left(t\right)=\bar{{\text{CF}}_{\text{rest}}}\;\left(\text{for a CF}\right), \end{array}$$

where $\bar{{\text{MF}}_{\text{rest}}}$ and $\bar{{\text{CF}}_{\text{rest}}}$ are set at 5 spikes/s and 1 spikes/s, respectively.

Once we define the firing rate of MF and CF as above, and assume that the activity of a simulated neuron (e.g., firing rate) reflects the strength of input signals to the neuron almost linearly as in the case of integrate-and-fire neurons used in this study (Gerstner et al., 2014), we could estimate the activity of VN as a linear sum of excitatory MF activity and inhibitory PC activity. The PC activity could be estimated as a linear sum of PF activity and basket cell activity, and further by solely MF activity. By substituting the MF and VN activities for MF(*t*) and VN(*t*) in Equation (4), we could obtain the following simplified equation for *v*. The detailed derivation is found in our previous paper (Yamazaki et al., 2015).

(7) $$\begin{array}{l} \tau_{v}\frac{dv}{dt}=-w\left(t\right)+w_{c}, \end{array}$$

where *w*(*t*) is the average synaptic weight of all PF-PC synapses, and *w*_*c*_ is a constant that defines the initial weight of PF-PC synapses, namely, 1.0. We used Equation (7) rather than Equation (4) for simplicity to update *v*(*t*).

### 2.3. Data analysis

We conducted computer simulation of the 5-days OKR training, and obtained spike data of all individual neurons and synaptic weight data of PF-PC synapses and MF-VN synapses. The total simulation time was 5 × 24 × 60 × 60 × 1000 = 4.32 × 10^8^ ms, with temporal resolution of 1 ms.

We analyzed how the OKR gain changed before and after training for each day. To do so, before training for each day, we fed 10 cycles of simulated optokinetic stimulus to the network and obtained the spike data of VN. We made a spike histogram with bin size of 100 ms, fitted the data with a cosine function with the period of 6 s, and calculated the modulation amplitude. We defined the modulation amplitude as the OKR gain before training. We made the same procedure to obtain the OKR gain after training for each day.

We also examined how the granule cells transmit mossy fiber signals robustly against noise in Poisson spike trains. Granule cells must produce almost identical spike pattern in response to the same optokinetic stimulus with different noise across cycles; otherwise, learning at Purkinje cells would fail. To quantify the reproducibility of the granule cell spike pattern in response to the same simulated optokinetic stimulus, we calculated the reproducibility index at time *t* defined as the normalized cross correlation as follows:

(8) $$\begin{array}{l} R\left(t\right)=\frac{\sum_{j}z_{j}^{\left(i\right)}\left(t\right)z_{j}^{\left(i+1\right)}\left(t\right)}{\sqrt{\sum_{j}z_{j}^{\left(i\right)}\left(t\right)}\sqrt{\sum_{j}z_{j}^{\left(i+1\right)}\left(t\right)}}, \end{array}$$

where $z_{j}^{\left(i\right)}\left(t\right)$ is the activity of granule cell *j* at cycle *i* of simulated optokinetic stimulus at time *t*, which was calculated by convolution of the spikes with a causal exponential:

(9) $$\begin{array}{l} z_{j}^{\left(i\right)}\left(t\right)=\underset{f\in S_{j}^{\left(i\right)}}{\sum }\exp\left(-\frac{t-t^{\left(f\right)}}{\tau }\right)\Theta \left(t-t^{\left(f\right)}\right), \end{array}$$

where $S_{j}^{\left(i\right)}$ is the set of spikes elicited by granule cell *j* at cycle *i*, *t*^(*f*)^ is the spike time for the *f*th spike, τ is the time constant of 8.3 ms, and Θ(*t*) is the Heaviside step function. Intuitively, $z_{j}^{\left(i\right)}\left(t\right)$ is a temporal trace of EPSPs of PF *j* on a PC at cycle *i*, and τ = 8.3 ms is the time constant of AMPA receptor-mediated PF-EPSPs at a PC (Llano et al., 1991). We calculated the average and standard deviation of the reproducibility index among 10 pairs of two successive cycles.

### 2.4. Numerical method

All equations that govern the network dynamics are solved numerically. Specifically, differential equations describing membrane potentials are solved by 2nd-order Runge-Kutta method with temporal resolution (Δ*t*) of 1 ms. The simulation program is written in C with CUDA (Common Unified Device Architecture) (NVIDIA, 2015) and most of the calculation is made on GPUs.

In our previous study (Yamazaki and Igarashi, 2013), we used only 1 GPU (NVIDIA GeForce GTX580) to simulate 100,000 granule cells in realtime. On the other hand, the present model has 10 times more granule cells, which makes computer simulation far slower than realtime. The most time-consuming part is to calculate synaptic conductances of Golgi cells, basket cells and PCs, where these cells receive excitatory inputs from granule cells via PFs. Due to the large number of granule cells, the calculation spends too much time. To address this issue, we decomposed the granular layer network composed of granule cells and Golgi cells into 4 identical subnetworks and calculated the dynamics in parallel on 4 GPUs (2 boards of NVIDIA GeForce GTX TITAN Z, each contains 2 GPUs). In the following, we explain how to decompose the network and calculate the conductance on 4 GPUs.

**Figure 2:**
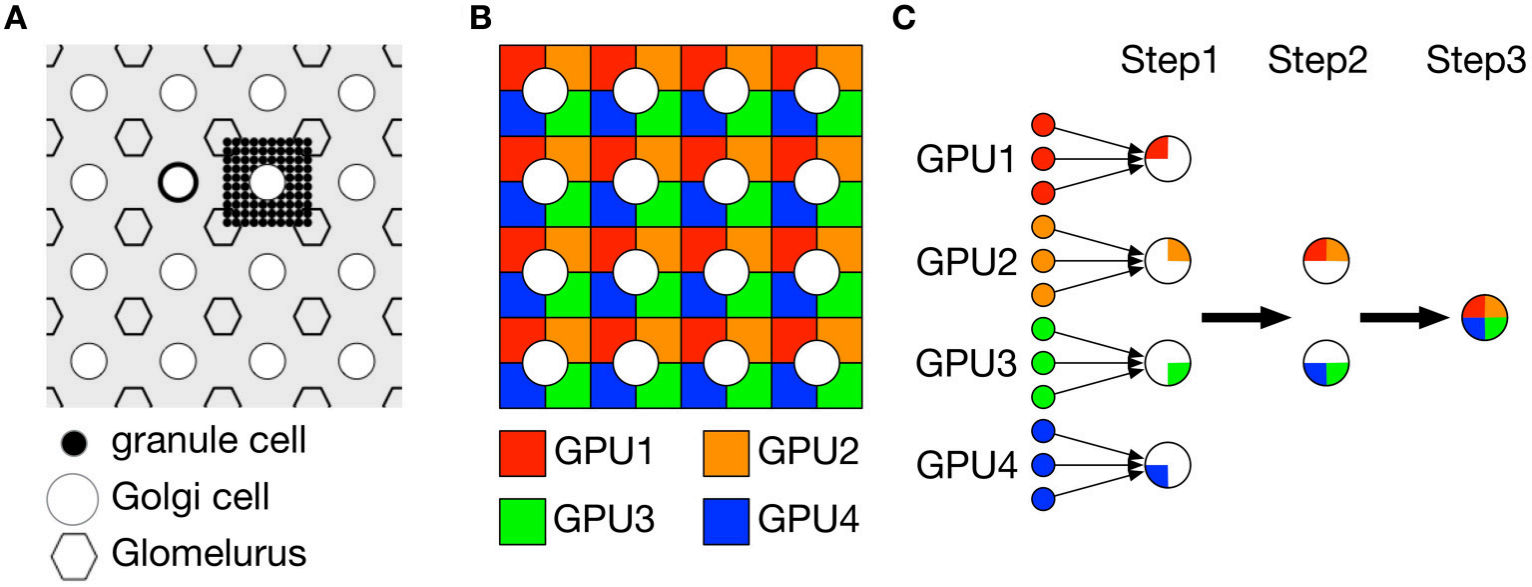
**Decomposition of granule cell population into four subpopulations for parallel simulation on 4 GPUs**. **(A)** Schematic of the model granular layer. Granule cells (black dot), Golgi cells (circle) and fictious glomeluri (hexagon) were arranged regularly on a two-dimensional grid. Each Golgi cell was surrounded by 4 glomeluri, and within the rectangle, 32 × 32 = 1024 granule cells were located and constituted a granule-cell cluster. These granule cells were assumed to share the same inhibitory inputs from Golgi cells and receive excitatory inputs for 4 independent mossy fibers, so the granule cells were functionally identical. **(B)** Decomposition of granule-cell clusters. We decomposed each granule-cell cluster composed of 32 × 32 = 1024 granule cells into 4 subclusters composed of 16 × 16 = 256 granule cells shown by 4 colors. **(C)** Calculation of synaptic conductances for Golgi cells, PCs and basket cells from granule cells (left small dots). Each postsynaptic neuron must sum up the postsynaptic potentials of all granule cells with certain synaptic weights. At step 1, for each postsynaptic neuron, a quarter of the synaptic conductance was calculated from granule cells on each GPU, which was illustrated by a pie-shape color where a large circle represents a postsynaptic neuron. At step 2, the calculated partial conductances were reduced between 2 GPUs in parallel to obtain a half of the conductance. At step 3, the partial conductances were further reduced and the full conductance was calculated. **(A)** was reproduced from Yamazaki and Tanaka (2007).

Figure 2A illustrates a part of the granular layer of our model. The granular layer is composed of 1024 × 1024 granule cells and 32 × 32 Golgi cells arranged regularly on a two-dimensional grid. Granule cells are further divided as 32 × 32 clusters, where each cluster consists of 32 × 32 granule cells. Due to short dendrites of granule cells, we assumed that the granule cells in the same cluster shared inhibitory inputs from the same Golgi cells. On the other hand, granule cells receive 4 excitatory MF inputs. We assumed that granule cells receive 4 MF inputs independently of the other granule cells. This structure allows us to decompose the granular layer network into 4 identical subnetworks composed of 512 × 512 granule cells and 32 × 32 Golgi cells, where granule cells are further divided into 32 × 32 clusters in which each cluster contained 16 × 16 = 256 granule cells as shown in Figure 2B. We conducted simulation of each subnetwork on a GPU, thereby we employed 4 GPUs for simulation of 4 subnetworks. In each subnetwork, we calculated quarter of synaptic conductance for each Golgi cell from granule cells in the same subnetwork. We then exchanged the partial conductances across subnetworks over GPUs and obtained the full conductance by summing up the partial values (Figure 2C). This is made by direct memory exchange between 2 GPUs called peer access, which is much faster than conventional memory exchange via CPUs. Because calculation of synaptic conductance is linear, our split-reduction method over 4 GPUs provides the same result with the conventional method. The same method is used to calculate synaptic conductances of basket cells and PCs as well.

## 3. Results

### 3.1. Simulation time

First, we measured how the simulation time was accelerated by using multi GPUs. Using only 1 GPU, we found that computer simulation of the cerebellar activity for 6 s, corresponding to 1 cycle of simulated optokinetic stimulus, spends 17.7 s. Using 2 GPUs, 9.10 s are spent. Finally, using 4 GPUs, we achieved 5.33 s for 6 s simulation, indicating realtime simulation. Therefore, we used 4 GPUs for further simulation.

### 3.2. Long-term OKR gain change

We conducted computer simulation of long-term OKR adaptation for 5 days. For each day, we performed a simulated 1-h OKR training. During the training, MFs convey simulated optokinetic stimuli, whereas a CF conveys simulated retinal slip error signals. After the training, both MFs and the CF elicit Poisson spikes spontaneously with a constant firing rate, respectively.

**Figure 3:**
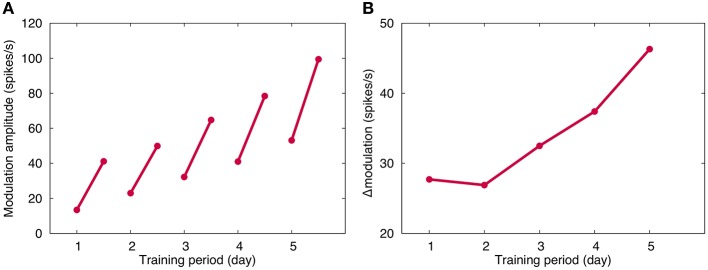
**Simulated OKR gain**. **(A)** Modulation amplitude of VN, which corresponded to OKR gain. Horizontal axis represents time (day) and vertical axis the firing rate (spikes/s). For each day, the modulation amplitude increased by 1-h training, and decreased after training until the next training in the next day. Throughout the 5 days, the amplitude gradually increased, suggesting consolidation of memory of learned OKR gain. **(B)** Increase of OKR gain before and after daily 1-h training. Conventions as in **(A)**. Although the same 1-h training was performed, the increase became larger day by day.

Figure 3A plots the OKR gain obtained in our long-term OKR training simulation for 5 days. By daily 1-h training, the OKR gain increases during training, and after the training, the learned OKR gain almost disappears. This indicates memory acquisition of OKR gain. On the other hand, throughout the 5 days, OKR gain gradually increases, indicating memory consolidation. The present numerical result is qualitatively consistent with previous experimental and theoretical results (Shutoh et al., 2006; Yamazaki et al., 2015).

Figure 3B plots the daily increment of learned OKR gain by 1-h training. The increment becomes larger day by day, suggesting that repeated daily training accelerates the memory acquisition. This result is consistent with previous experiments (Shutoh et al., 2006).

### 3.3. Change of synaptic weights

**Figure 4:**
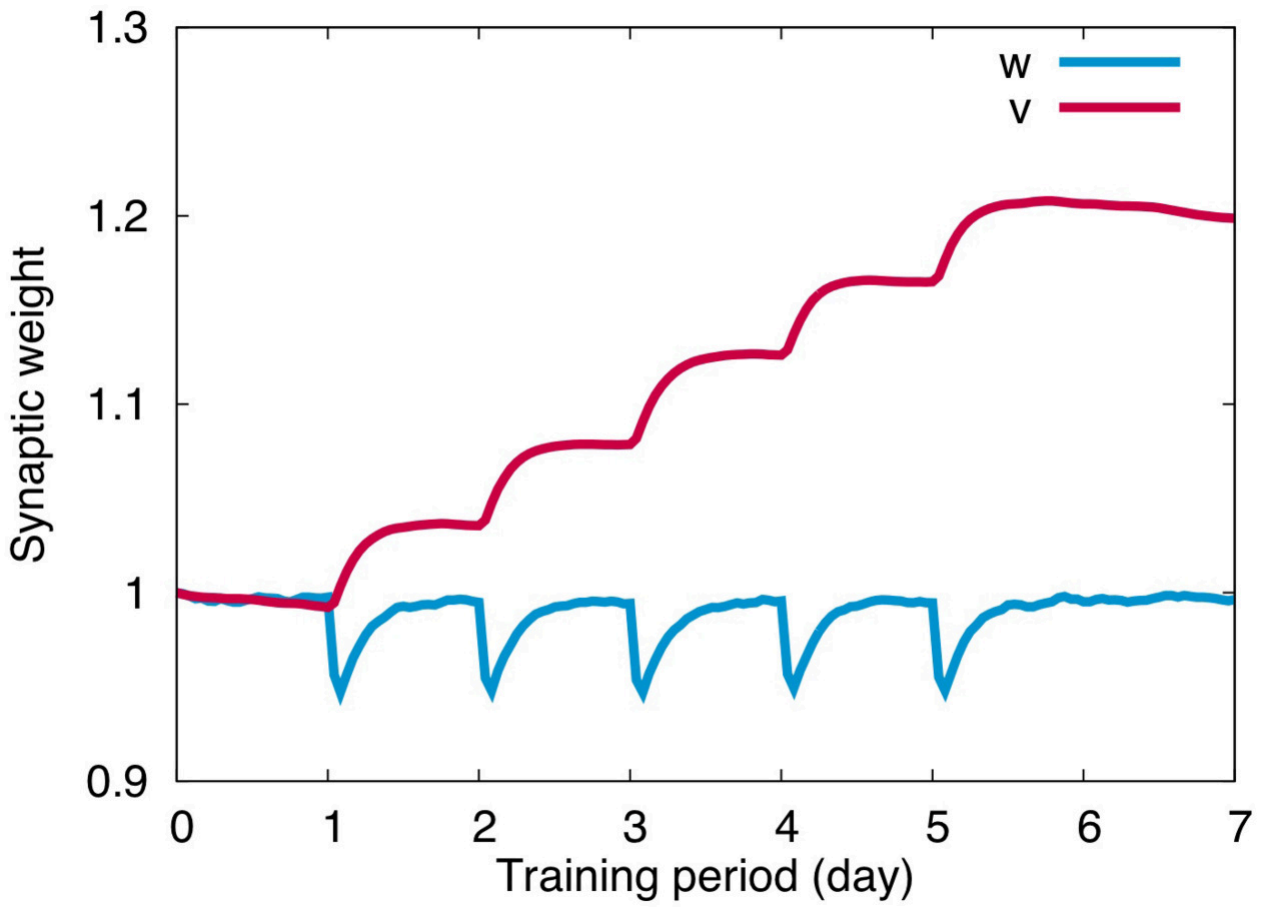
**Synaptic weight change**. PF-PC synaptic weights (blue) repeated quick decrease during training and slow recovery after training for each day, whereas MF-VN synaptic weight started to increase mainly after training and was accumulated throughout 5 days. Horizontal axis represents training period (day) and the vertical axis represents the weight value. For PF-PC synaptic weights, the average value on all presynaptic granule cells and postsynaptic PCs was plotted.

Figure 4 plots the change of weights at PF-PC synapses (*w*) and MF-VN synapses (*v*) throughout the 5 days training. For *w*, we calculated the average of all PF-PC synaptic weights with respect to PFs and PCs. Similarly for *v*, we calculated the average of all MF-VN synaptic weights with respect to MFs. PF-PC synapses undergo LTD during training, and slowly return to the original weight value after training spontaneously. PF-PC synapses repeat the same temporal change 5 times for 5 days, suggesting that PF-PC synapses store only short-term memory of OKR gain for hours. On the other hand, MF-VN synapses change little during training, and slowly increase after training. The synaptic weight accumulates every day after training, suggesting that MF-VN synapses store long-term memory of OKR gain.

The overall dynamics is as follows. First, memory of OKR gain is formed in the cerebellar cortex by PF-PC LTD during training. Second, after training, learned cortical memory is decayed slowly and disappears completely by the next day, and finally, during the slow decay of the cortical memory, memory is formed in the vestibular nucleus by MF-VN LTP, as if the cortical memory is transferred to the nucleus and consolidated. The present numerical result is consistent with the previous theoretical results (Yamazaki et al., 2015).

### 3.4. Change of eye movement trajectory

So far, both the current numerical and previous theoretical studies show qualitatively the same results. A benefit of our numerical study is that we could obtain detailed data of individual neurons such as membrane potential and spike trains with a fine temporal resolution of 1 ms, which were abstracted in our theoretical model (Yamazaki et al., 2015).

**Figure 5:**
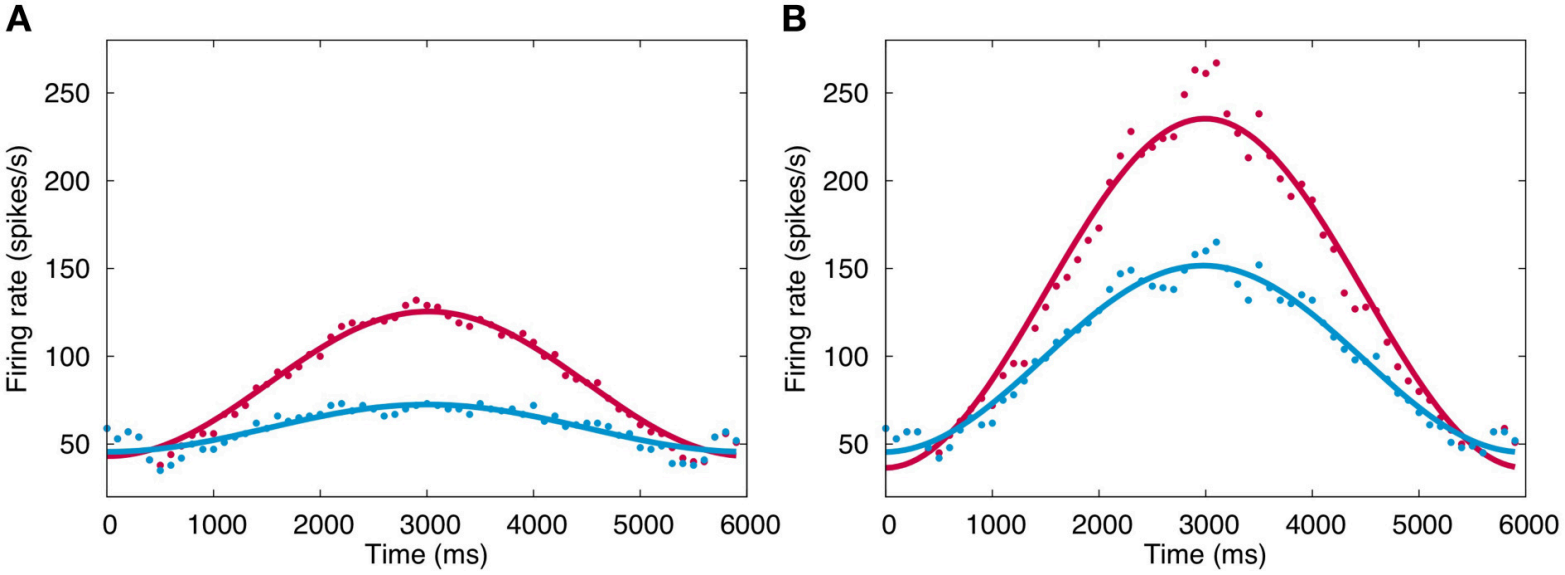
**Firing rate change of VN**. **(A)** Firing rate in response to simulated optokinetic stimulus modulating sinusoidally in time with the period of 6 s before and after training (blue and red, respectively) in the 1st day. Horizontal axis represents time in ms, and vertical axis the firing rate (spikes/s). The data points are fitted with a cosine function and the fitted curves are also plotted to show the modulation clearly. **(B)** The same firing rate in the 5th day. Conventions as in **(A)**.

Figure 5 plots the firing rate of VN in response to simulated sinusoidal optokinetic stimulus before and after training at the 1st day (A) and the 5th day (B). The firing rate modulates sinusoidally as the input signals. The modulation amplitude increases by daily 1-h training, and the amplitude also increases gradually throughout 5 days. On the other hand, the baseline firing rate does not change largely from 30–50 spikes/s. Here, the modulation amplitude of VN represents the OKR gain (Shutoh et al., 2006), suggesting that the OKR gain becomes larger by repeated daily training. These results also suggest that realtime simulation allows us to study both macroscopic behaviors of a neural network such as OKR gain, and mesoscopic dynamics of individual neurons in the neural network such as a membrane potential and spike trains.

### 3.5. Robust signal transmission by the enormous number of granule cells

Granule cells must transmit information conveyed by mossy fibers to Purkinje cells and interneurons faithfully against input noise, otherwise, learning at Purkinje cells would fail. In OKR, mossy fibers convey information on visual world movement, and granule cells produce a spatiotemporal spike pattern that represents the stimulus reliably. For this purpose, the almost identical spike pattern of granule cells must be produced across cycles of the optokinetic stimulus.

Here, we examined how the enormous number of granule cells help them to transmit mossy fiber information faithfully and robustly against input noise. Specifically, we calculated the reproducibility index (Equation 8) that quantifies the reproducibility of the spike pattern of granule cells across cycles of the simulated optokinetic stimulus on different cycles, while changing the number of granule cells in the network.

**Figure 6:**
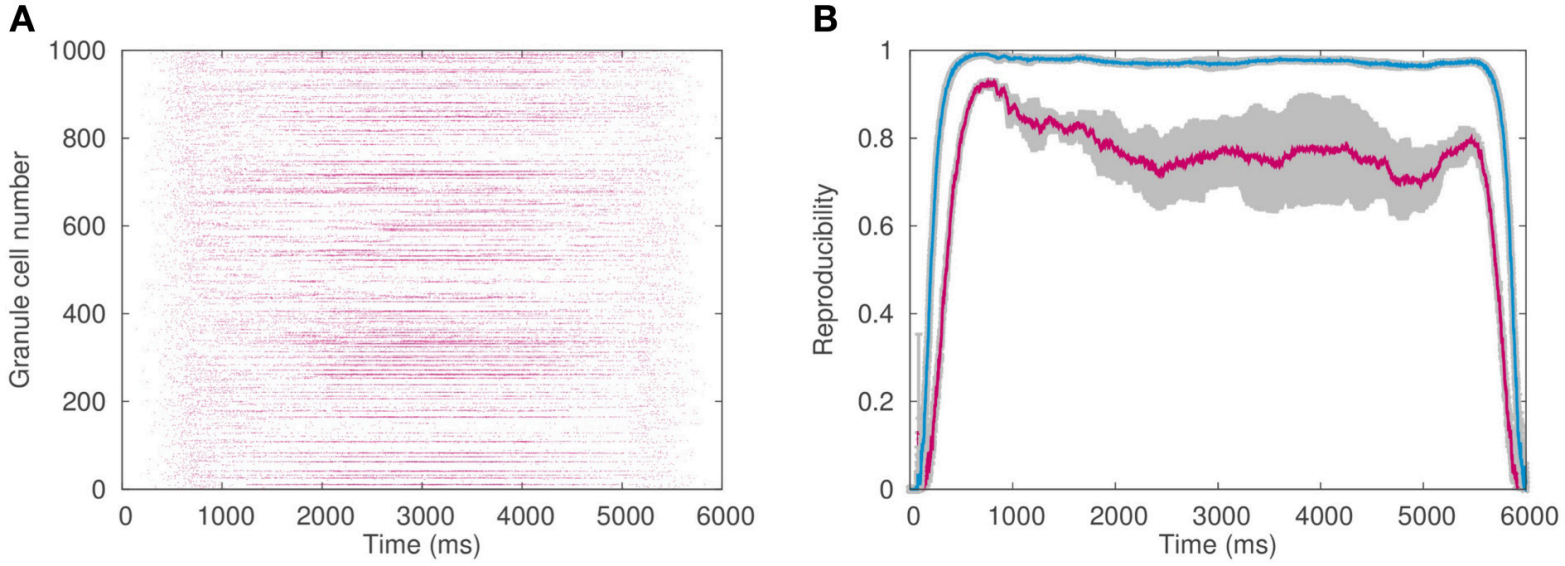
**Granule cell activity**. **(A)** Spike pattern of 1024 out of 1,048,576 granule cells chosen randomly in response to simulated optokinetic stimulus, which modulated sinusoidally with the period of 6 s. Horizontal axis represents time (ms), whereas the vertical axis the neuron number. **(B)** Reproducibility of the granule-cell spike pattern across different cycles of the simulated stimulus. The reproducibility with 1 million granule cells (blue) was higher than that with 0.1 million cells (red). Horizontal axis represents time (ms), whereas the vertical axis the reproducibility index. The average values on 10 pairs of cycles were plotted in color, while gray regions represented the standard deviation.

Figure 6A plots an example of the spike pattern of granule cells, whereas Figure 6B plots the reproducibility. As can be seen, the reproducibility is better when 1 million granule cells were employed than 0.1 million granule cells. This result suggests that a functional role of the enormous number of granule cells is robust transmission of mossy fiber signals to PCs against input noise.

## 4. Discussion

### 4.1. Understanding memory consolidation mechanisms

Memory consolidation is a slow process that takes days and weeks. To study the neural mechanisms of memory consolidation, two ways are possible: either conducting experiments or making theoretical models. A theoretical model is a mathematical description of a specific phenomenon. To make such model, we ignore most of experimental details and capture the essence of the phenomenon. For example, in our theoretical model of posttraining memory consolidation in the cerebellum (Yamazaki et al., 2015), we abstracted all detailed physiology of individual neurons, detailed anatomical structure, and detailed input stimuli. This provides a clear view of how the memory consolidates after training, but we still do not know the detailed neuronal process during the memory consolidation. Large-scale, realistic spiking network models are appropriate for this purpose, but the computational time would be problematic instead.

HPC technology solves this problem. The advantage is two-folds. First, the technology allows us to build a larger-scale model composed of more neurons and synapses with more detailed morphology and biophysical properties than conventional models. Very large-scale functional brain models have been built (Izhikevich and Edelman, 2008; Eliasmith et al., 2012). Notably, The Blue Brain Project and Human Brain Project attempt to build a realistic whole brain model, and they recently published a very detailed cortical microcolumn model (Markram et al., 2015). Second, the technology allows us to carry out computer simulations much faster than that on a single-threaded CPU. The latter makes the above-mentioned long-term computer simulation possible in a reasonable time. For instance, if the computer simulation runs in real time, a simulation of memory consolidation for 1 week completes in 1 week. In our study, we adopted GPUs. Using our large-scale, detailed spiking network model of the cerebellum implemented on multi GPUs, we were able to simulate the detailed temporal dynamics of individual neurons, while observing the slow memory consolidation process simultaneously. The present study is, as far as the author knows, a first demonstration of a very long time computer simulation of an elaborated spiking network model for days. We were able to replicate our previous theoretical results (Yamazaki et al., 2015), and further examined detailed neuronal and synaptic dynamics during memory consolidation. In cerebellar motor learning, location of motor memory and the role of LTD at PF-PC synapses have been a matter of debate for more than 30 years (Mellvill-Jones, 2000). The present study could provide an answer from the modeling view point.

### 4.2. Realtime simulation and the programming

The present cerebellar model consists of more than 1 million spiking neurons. In general, computer simulation of such large-scale model takes very long time. The simulation could be 10–100 times slower than the real-world time (Nageswaran et al., 2009). However, owing to HPC technology, we were able to conduct computer simulation in realtime, where simulation of cerebellar activity for 1 s completes within 1 s in the real-world time. This allowed us to conduct a complete computer simulation of long-term OKR adaptation training for 5 days in a practical time.

We used 4 GPUs simultaneously to perform realtime simulation of 1 million neurons. To do so, we had to write the simulation program in C with CUDA, a platform for GPU computing, and employed some parallel algorithms to use GPUs efficiently. Specifically, we used some algorithms to compute synaptic conductances of Golgi cells, basket cells and PCs that receive excitatory inputs from many granule cells. This is quite technical and difficult, and so there should be a more simple way to adopt the power of parallel computing in neuroscience. One potential way would be to develop a neural simulator primarily designed for GPUs and some other accelerators. Naveros et al. (2014) has reported development of such a spiking neuron network simulator on a GPU. Some groups have used the software for realtime robot control (Garrido et al., 2013; Casellato et al., 2015).

Realtime simulation is only a milestone, and we expect even faster computer simulation. Scalability, however, would be a problem. Generally speaking, using more GPUs would employ more latency for communications and overhead of communication operations, which could easily be a bottle neck.

### 4.3. Advantages of large-scale models over theoretical models

Although the present study reproduced qualitatively the same results with our previous theoretical model (Yamazaki et al., 2015), some results are slightly different. First, the MF-VN synaptic weight in the present model tends to decay spontaneously, whereas that in the theoretical model did not. This is because in the theoretical model, the decay term was canceled out and removed by a mathematical treatment. In experiments (Shutoh et al., 2006), the learned long-term OKR gain almost vanishes after 2 weeks from the last training, suggesting that it is natural for the synaptic weight to decay spontaneously. Second, the increase of modulation amplitude of VN before and after the 1-h training gradually becomes larger throughout 5 days in the current study (Figure 2B), whereas the change is constant in the theoretical model. The same experiments demonstrate that the increase becomes larger gradually day by day. This result suggests that the present large-scale model captures the detailed dynamics of long-term OKR gain adaptation better than the theoretical model.

Moreover, the present model allows us to study the detailed temporal dynamics of individual neurons with a fine temporal resolution. We were able to obtain detailed spike data of PCs and VN, and analyzed the firing patterns as in Figure 6. This is an advantage of an elaborated spiking network model over theoretical models, which abstract detailed temporal dynamics of individual neurons. We will be able to go into the details of molecular mechanisms of memory acquisition and consolidation (Abel and Lattal, 2001; Ito, 2002), if the HPC technology advances further.

We were also able to examine how the number of neurons could affect the stability of the network dynamics. In the present model, we incorporated more than 1 million granule cells, because the cats' cerebellum has 1 million granule cells per 1 mm^3^ (Ito, 1984). The cerebellar granule cells constitute the largest population in the whole brain (Azevedo et al., 2009). A question arises: why does the cerebellum have such an enormous number of granule cells? A theoretical study has demonstrated that incorporating more granule cells makes the network more reliable for controlling hardware robots (Pinzon-Morales and Hirata, 2015). In the present study, we demonstrated that the enormous number of granule cells makes signal transmission from MFs to PFs more robust as in Figure 6.

In summary, combination of large-scale, detailed spiking network models with HPC technology for realtime simulation will provide a strong means to study mesoscopic, detailed neural mechanisms for macroscopic behavioral phenomenon that could take very long time for days and weeks such as memory formation.

### 4.4. Data sharing

We will release the source code of the model used in this study under an opensource license upon publication, to facilitate open collaboration and ensure scientific reproducibility, on Cerebellar Platform (https://cerebellum.neuroinf.jp/).

## Author contributions

TY designed research; MS and TY performed research; MS and TY analyzed data; TY and MS wrote the paper.

## Funding

JSPS Kakenhi Grant Number (26430009) and UEC Tenure Track Program (6F15).

### Conflict of interest statement

The authors declare that the research was conducted in the absence of any commercial or financial relationships that could be construed as a potential conflict of interest.
